# Large‐Scale Implementation of Vertical Sidewall and Vertical Multi‐Channel WS_2_ Nanosheet Field‐Effect Transistors for Area‐Efficient Integrated Circuit

**DOI:** 10.1002/smll.202508533

**Published:** 2025-09-02

**Authors:** Jiwon Ma, Eunyeong Yang, Changwook Lee, Jisoo Seok, Jiwon Chang

**Affiliations:** ^1^ Department of System Semiconductor Engineering and Department of Materials Science and Engineering Yonsei University Seoul 03722 South Korea

**Keywords:** 2D materials, area‐efficient integrated circuit, double‐gate, multi‐channel nanosheet field‐effect transistor, tungsten disulfide, vertical channel field‐effect transistor, vertical sidewall field‐effect transistor

## Abstract

2D materials have emerged as promising candidates for next‐generation field‐effect transistors (FETs) owing to the atomically thin geometry and excellent electrostatic gate control. Here, double‐gate vertical sidewall FETs based on chemical vapor deposition‐grown monolayer WS_2_ are demonstrated and, for the first time, report vertical multi‐channel nanosheet FETs (NSFETs). By implementing a dual‐step sidewall profile, steep SiO_2_ surfaces are obtained, which enabled seamless WS_2_ adhesion and contributed to enhanced device yield. The fabricated vertical sidewall WS_2_ FETs exhibited good subthreshold swing (SS) and effectively suppressed short‐channel effects at channel length as short as 150 nm. Logic gates including inverters, NAND, NOR, AND, OR, and SRAM are integrated using vertical sidewall and planar WS_2_ FETs, validating the feasibility of area‐efficient integrated circuit. Furthermore, improved drive current is achieved in vertical multi‐channel NSFETs realized by stacking WS_2_ channels and employing a gate‐all‐around–like structure. These results highlight the potential of vertical sidewall FETs for enabling area‐efficient, ultra‐dense integrated circuits.

## Graphical abstract

In this work, the authors successfully demonstrate double‐gate vertical sidewall WS_2_ FETs and, for the first time, vertical multi‐channel WS_2_ NSFETs using CVD‐grown monolayer WS_2_. By optimizing dual‐step SiO_2_ sidewall profiles and employing high‐κ dielectrics, they achieve excellent subthreshold swing, suppressed short‐channel effects, and high *I*_ON_/*I*_OFF_ ratios down to 150 nm channel lengths. Furthermore, area‐efficient logic gates and SRAM circuits are realized by integrating vertical sidewall and planar WS_2_ FETs, highlighting the scalability and potential of vertical 2D FET architectures for ultra‐dense integrated circuits.

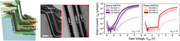

## 1 Introduction

As semiconductor technologies approach the physical scaling limits, 2D materials, such as molybdenum disulfide (MoS_2_) and tungsten disulfide (WS_2_) have emerged as promising channel materials for ultra‐scaled field‐effect transistors (FETs).^[^ ^1^, ^2^ ^]^ The atomically thin geometry of 2D materials enables excellent electrostatic gate control over the channel, which effectively suppresses short‐channel effects (SCEs).^[^ ^3^, ^4^, ^5^ ^]^ Among various 2D materials, monolayer MoS_2_ and monolayer WS_2_ offer relatively large bandgaps and effective masses, which help suppress source‐to‐drain tunneling and minimize off‐state leakage—critical advantages for low power logic applications.^[^ ^6^, ^7^ ^]^ In addition to the electrical properties advantageous in an aggressively scaled regime, the ultra‐thin nature of 2D materials makes them highly suitable for integration into non‐planar, vertically channel FETs where the current conduction occurs along the vertically aligned channel.^[^ ^8^, ^9^, ^10^, ^11^, ^12^, ^13^ ^]^ Vertical channel FETs provide improved area efficiency and scalability for ultra‐dense integrated circuits.^[^ ^14^ ^]^ Compared to conventional planar channel FETs, vertical channel FETs can significantly reduce the device footprint by forming the channel in the vertical direction. Furthermore, channel length (*L*_CH_) can be extended in the vertical direction to effectively suppress SCEs without sacrificing area efficiency.

Recent studies have demonstrated the integration of 2D materials into vertical channel FETs. Jia et al.^[^ ^10^ ^]^ reported the fabrication of MoS_2_ vertical channel FETs using an etched sidewall structure. However, the structure exhibited a relatively shallow sidewall angle of ≈30–40°, which limits the area scaling for high‐density integration. Tao et al.^[^ ^11^ ^]^ explored stamping techniques for transferring MoS_2_ onto vertical facets, however, these approaches often suffer from poor alignment and lack scalability, making them less viable for practical large‐area applications. In this work, we present vertical sidewall FETs based on chemical vapor deposition (CVD)‐grown monolayer WS_2_. Our vertical sidewall WS_2_ FETs was fabricated on the etched SiO_2_ sidewall with a steep angle (≈70°), aiming to maximize the area efficiency. Through fine tuning of the SiO_2_ etching process, we achieved an optimal SiO_2_ etching profile to reduce strain at the sidewall corner and improve the adhesion of monolayer WS_2_, which eliminated interfacial air gaps and consequently increased device yield while reducing device‐to‐device variability. Through the introduction of high‐*k* dielectric and double‐gate (DG) structure, *L*_CH_ scaling was achieved with minimal degradation in subthreshold slope (*SS*), and the on/off current (*I*_ON_/*I*_OFF_) ratio. By combining vertical sidewall FETs with planar FETs, we successfully demonstrated nMOS logic gates such as inverters, NAND, NOR, AND, OR gates, and SRAM. Furthermore, the vertical sidewall FETs architecture was extended into vertical multi‐channel nanosheet (NS) FETs by stacking WS_2_ channels. The adoption of multi‐channel with a gate‐all‐around‐like structure enhances both drive current and gate control, presenting the first experimental demonstration of vertical multi‐channel NSFETs based on 2D materials.

## 2 Results and Discussion

### 2.1 Optimization of SiO_2_ Sidewall Profiles for Vertical Sidewall FETs

**Figure 1:**
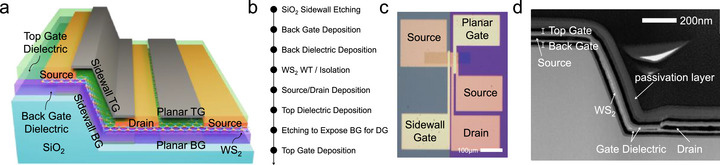
Structure and fabrication process of DG vertical sidewall and planar WS_2_ FETs. a) Schematic illustrations of DG vertical sidewall and planar WS_2_ FETs. b) Fabrication process of DG vertical sidewall and planar FETs based on CVD‐grown monolayer WS_2_. c) Optical microscopy image showing the fabricated vertical sidewall and planar FETs. d) Cross‐sectional TEM image of vertical sidewall FETs, confirming that the overall device geometry is successfully realized.

**Figure** **1**a illustrates our DG vertical sidewall FETs integrated with planer FETs for nMOS inverter. As shown in the fabrication process of Figure 1b, first, a SiO_2_ sidewall was formed via dry etching. Vertical sidewall FETs was subsequently fabricated along the SiO_2_ sidewalls, while planar FETs was formed on the etched planar SiO_2_ surface. An optical microscopy image of the fabricated vertical sidewall FETs and planar FETs is presented in Figure 1c. To enhance the performance of sidewall FETs, various dry etching conditions were explored to achieve the optimal SiO_2_ sidewall profile. The full dry etching parameters—including gas composition, RF power, pressure, and mask materials—used to form SiO_2_ sidewalls under three representative conditions are summarized in Table S1 (Supporting Information). For each etching condition, we fabricated sidewall FETs using monolayer MoS_2_ as the channel. Notably, monolayer MoS_2_ was employed only for optimizing the sidewall etching condition, while monolayer WS_2_ was utilized in the other parts of the study to explore the performance of vertical sidewall FETs and to implement the logic gates and vertical multi‐channel NSFETs. Since MoS_2_ and WS_2_ share the same 2H hexagonal crystal structure, similar mechanical behavior is expected when transferred onto the etched SiO_2_ sidewall geometry. Notably, the reported elastic strain limit of MoS_2_ (≈6%–11%)^[^ ^15^ ^]^ is lower than that of WS_2_ (≈15.5%),^[^ ^16^ ^]^ suggesting that if MoS_2_ maintains structural stability on the sidewall topology, particularly at the corner regions, WS_2_ is expected to exhibit comparable or superior mechanical stability under identical conditions.

In sidewall MoS_2_ FETs, 20 nm Al_2_O_3_ was used as the back‐gate dielectric. For the top‐gate dielectric, a 1.5 nm aluminum (Al) seed layer was first deposited, followed by the deposition of 16 nm Al_2_O_3_. The *L*_CH_ of sidewall MoS_2_ FETs was fixed at 700 nm for all three types of sidewalls, while only the profile of the SiO_2_ sidewall was varied to investigate the effect on device performance. For the first etching condition, a high etch rate resulted in a steep sidewall profile^[^ ^17^ ^]^ with an angle of ≈75°, as shown in Figure S1a (Supporting Information). Since the nickel hard mask^[^ ^18^ ^]^ resulted in a steep sidewall profile, 2D film exhibited poor adhesion to the sidewall, leading to the formation of air gaps at the corner^[^ ^19^, ^20^ ^]^ as shown in Figure S2 (Supporting Information). The observed poor adhesion at the bottom corner can be understood from a strain perspective. Due to the steep sidewall profile of the SiO_2_ substrate, the local curvature radius at the bottom edge is reduced to approximately *R* = 4.91 nm.

(1) $$\varepsilon =\frac{t}{2R}$$

According to Equation (1), this induces a strain of ≈6.62% in the 2D channel, which approaches the theoretical elastic strain limit^[^ ^15^, ^21^, ^22^ ^]^ of monolayer MoS_2_. To mitigate excessive elastic strain induced by the sharp corner geometry, the 2D channel may locally delaminate and form an air gap,^[^ ^23^, ^24^, ^25^ ^]^ as the system minimizes the total free energy by relaxing strain at the cost of reduced adhesion. This air gap resulted in very low device yield and substantial device‐to‐device variation in the transfer (*I*_DS_–*V*_GS_) characteristics, as plotted in Figure S1b (Supporting Information). Moreover, we observed poor gate control over the channel, as reflected in the degraded *I*_ON_/*I*_OFF_ ratio. In the second etching condition, the inductively coupled plasma (ICP) power was lowered, thereby decreasing the etch rate^[^ ^26^ ^]^ and yielding a reduced sidewall angle of ≈70°, as shown in Figure S1c (Supporting Information). This adjustment improved device‐to‐device variation in the *I*_DS_–*V*_GS_ characteristics (Figure S1d, Supporting Information). However, gate control remained insufficient, leading to high *SS* and low *I*_ON_/*I*_OFF_ ratio. To address these challenges, dual‐step profile was introduced in the third etching condition to ensure good adhesion and eliminate air gaps between the 2D channel and the sidewall. As seen in Figure S1e (Supporting Information), the upper sidewall maintained a steep sidewall profile (≈70°), while the lower part exhibited a much lower slope (≈10°). Unlike the previous etching conditions, a negative photoresist (PR) was used as the masking layer to enable dual‐step profile. The dual‐slope sidewall structure facilitated conformal adhesion of the wet‐transferred 2D film onto the sidewall, effectively preventing air gap formation, as confirmed in the cross‐sectional TEM images (Figure 1d; Figures S3 and S4, Supporting Information). The 2D channel is also subjected to strain when conformally transferred over the top and bottom corners of the structure. At the top corner, a curvature radius of approximately *R* = 72.23 nm results in a strain of ≈0.45% (Figure S3a, Supporting Information), while the bottom corner, with a curvature radius of *R* = 153.06 nm, induces a strain of ≈0.212% (Figure S3b,Supporting Information). These strain values are negligible compared to the theoretical elastic strain limits of monolayer 2D materials, confirming the mechanical and structural stability of the transferred configuration. Furthermore, EDS mapping presented in Figure S4 (Supporting Information) confirms the successful fabrication of DG WS_2_ FETs on the sidewall. Cross‐sectional TEM images reveal significant overlap between the gate and the source/drain electrodes, which is intentionally designed to ensure full coverage of the channel region by the gate, thereby maximizing the gating efficiency. The improved sidewall profile led to excellent gate control in the *I*_DS_–*V*_GS_ characteristics (Figure S1f, Supporting Information), with the reduced *SS* and enhanced *I*_ON_/*I*_OFF_ ratio. Since the SiO_2_ etching process had a negligible effect on planar FETs, both *SS* and *I*_ON_/*I*_OFF_ ratio remained consistent for all three etching conditions, as presented in Figure S5 (Supporting Information). Based on these findings, the third etching condition was adopted for the fabrication of the remaining DG vertical sidewall WS_2_ FETs.

### 2.2 Channel Length and Dielectric Scaling in DG Vertical Sidewall WS_2_ FETs

**Figure 2:**
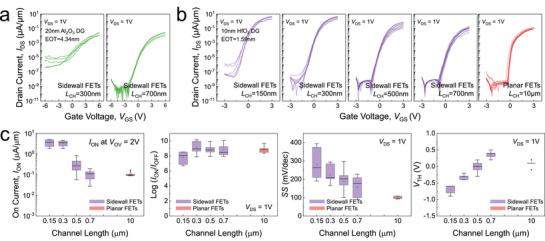
Electrical performance of DG vertical sidewall and planar WS_2_ FETs with different channel lengths and dielectric stacks. a) *I*_DS_–*V*_GS_ characteristics of vertical sidewall FETs with *L*_CH_ = 700 nm and 300 nm using Al_2_O_3_ for top‐ and back‐ gate dielectrics (EOT = 4.34 nm). b) *I*_DS_–*V*_GS_ characteristics of vertical sidewall and planar FETs using HfO_2_ and Al_2_O_3_ for back‐gate and top‐gate dielectrics, respectively, (EOT = 1.59 nm) with *L*_CH_ = 150–700 nm for vertical sidewall FETs and with *L*_CH_ =10 µm for planar FETs. c) Extracted *I*_ON_, *I*_ON_/*I*_OFF_ ratio, *SS*, and *V*_TH_ as a function of *L*_CH_ for vertical sidewall and planar FETs.

First, we fabricated DG vertical sidewall WS_2_ FETs on an optimized sidewall employing Al_2_O_3_ as the top‐ and back‐gate dielectrics. The back‐gate dielectric is 20 nm Al_2_O_3_, while the top‐gate dielectric comprises a 1.5 nm Al seed layer and 16 nm Al_2_O_3_, designed to induce optimal n‐type doping in the WS_2_ channel.^[^ ^27^ ^]^ To evaluate the impact of *L*_CH_, we investigated the performance of Al_2_O_3_ DG devices with *L*_CH_ values of 300 nm and 700 nm. Here, *L*_CH_ is defined as the distance between the source and drain electrodes, comprising both a vertical segment along the sidewall and a horizontal segment shorter than 100 nm. For statistical validation and reproducibility of measured data, we analyzed multiple devices for each channel length and presented the distribution of key metrics in boxplot form (**Figure** **2**c). The *I*_DS_–*V*_GS_ characteristics were measured by sweeping *V*_GS_ from –6 to 6 V in 0.1 V steps at *V*_DS_ = 1 V. The channel width (*W*) of all devices was 50 µm. As shown in Figure 2a, the device with *L*_CH_ = 700 nm exhibits *I*_DS_–*V*_GS_ characteristics with the excellent *I*_ON_/*I*_OFF_ ratio larger than 10^9^ and decent *SS* ≈230.8 mV/dec. However, in the device with *L*_CH_ = 300 nm, a significant increase in *I*_OFF_ is observed, resulting in a substantially reduced *I*_ON_/*I*_OFF_ ratio ≈10^4^. Additionally, *SS* is significantly degraded to ≈1000 mV dec^−1^, indicating severe SCEs.^[^ ^28^, ^29^ ^]^ To improve gate control over the channel in short‐channel devices, we scaled down the equivalent oxide thickness (EOT).^[^ ^30^, ^31^, ^32^ ^]^ The EOT in the DG configuration was calculated based on the following two equations:

(2) $$EOT=\frac{3.9}{\varepsilon_{dielectric}}\times t_{dielectric}$$

(3) $$\frac{1}{EOT_{double}}=\frac{1}{EOT_{top}}+\frac{1}{EOT_{back}}$$

**Figure 3:**
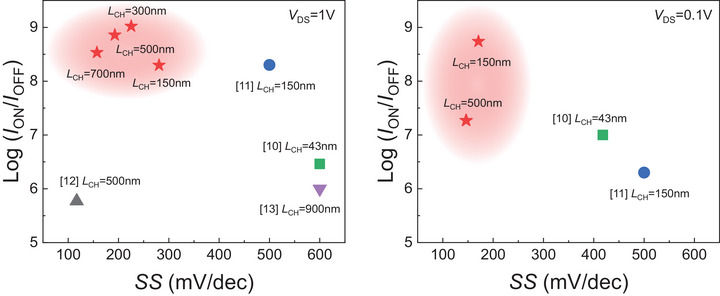
Benchmarking of key performance metrics between our DG vertical sidewall WS_2_ FETs and previously reported sidewall MoS_2_ FETs under two bias conditions: a) *V*_DS_ = 1 V and b) *V*_DS_ = 0.1 V. Our devices exhibit superior *I*_OFF_ suppression, enhanced *I*_ON_/*I*_OFF_ ratio, excellent *SS* highlighting their strong immunity to short‐channel effects and scalability advantages for low‐power logic applications.

To reduce EOT, the back‐gate dielectric was replaced with 10 nm HfO_2_ instead of 20 nm Al_2_O_3_. Meanwhile, the top‐gate dielectric, consisting of a 1.5 nm Al seed layer and 16 nm Al_2_O_3_, was retained to maintain efficient n‐type doping of the WS_2_ channel.^[^ ^27^ ^]^ With the introduction of HfO_2_, EOT was scaled down from 4.34 to 1.59 nm. Accordingly, *V*_GS_ sweep range for the *I*_DS_–*V*_GS_ measurements was reduced to a range of –3 to 3 V, while maintaining the same step size of 0.1 V. Figure 2b presents the *I*_DS_–*V*_GS_ characteristics of vertical sidewall FETs with *L*_CH_ values of 150, 300, 500, and 700 nm, as well as planar FETs (*L*_CH_ = 10 µm), with EOT = 1.59 nm at *V*_DS_ = 1 V. The *I*_DS_–*V*_GS_ characteristics at *V*_DS_ = 0.1 and 0.2 V are presented in Figure S6 (Supporting Information). We extracted *I*_ON_ at *V*_GS_ = *V*_TH_ + *V*_OV,_^[^ ^33^ ^]^ where the overdrive voltage (*V*_OV_) was set to 2 V, and the threshold voltage (*V*_TH_) was extracted using a reference current of 1 nA µm^−1^.^[^ ^34^, ^35^ ^]^ As *L*_CH_ is scaled down, *I*_ON_ increases from 0.11 to 3.68 µA µm^−1^ (Figure 2c) primarily due to the reduced channel resistance.^[^ ^35^, ^36^ ^]^ Planar FETs with *L*_CH_ = 10 µm exhibit much larger channel resistance compared to sidewall FETs. Consequently, *I*_ON_ of sidewall FETs with *L*_CH_ = 150 nm reaches 3.68 µA µm^−1^, whereas *I*_ON_ of planar FETs is only 0.061 µA µm^−1^, clearly demonstrating the impact of *L*_CH_ on the performance. *V*_TH_ roll‐off and *SS* degradation are clearly observed,^[^ ^37^, ^38^ ^]^ indicating severe SCEs. *SS* is ≈150 mV dec^−1^ at *L*_CH_ = 700 nm, but degrades to ≈300 mV dec^−1^ at *L*_CH_ = 150 nm. With respect to the *I*_ON_/*I*_OFF_ ratio, as *L*_CH_ decreases from 700 nm, the ratio initially improves since *I*_ON_ increases, while *I*_OFF_ remains ≈10^−9^ µA µm^−1^. However, at *L*_CH_ = 150 nm, slight SCEs lead to an increase in *I*_OFF_, which in turn degrades the *I*_ON_/*I*_OFF_ ratio down below 10^8^. Additionally, a slight increase in drain‐induced barrier lowering (DIBL) was observed as *L*_CH_ decreases.^[^ ^39^, ^40^ ^]^ DIBL was 118.8 mV V^−1^ for *L*_CH_ = 150 nm. Compared to the performance of Al_2_O_3_ DG devices in Figure 2a, the use of HfO_2_ results in significantly improved performance at *L*_CH_ = 300 nm and even at shorter channel length. **Table** **1** and **Figure** **3** present the performance benchmarking between our DG vertical sidewall WS_2_ FETs and previously reported sidewall MoS_2_ FETs under two bias conditions: *V*_DS_ = 1 V and *V*_DS_ = 0.1 V. Specifically, Figure 3 focuses on *SS* and the *I*_ON_/*I*_OFF_ ratio. At *V*_DS_ = 1 V (Figure 3a), our devices achieve superior *SS* and the highest *I*_ON_/*I*_OFF_ ratio compared to prior reports. Even under low‐bias conditions (*V*_DS_ = 0.1 V, Figure 3b), they maintain excellent *SS* and competitive *I*_ON_/*I*_OFF_ ratios.

**Table 1:** Performance Benchmarking of Vertical Sidewall FETs at *V*_DS_ = 1 and 0.1 V.

	References	Channel	*L*_CH_ [nm]	*I*_ON_ [µA µm^−1^]	*I*_OFF_ [µA µm^−1^]	*I*_ON_/*I*_OFF_	*SS* [mV dec^−1^]	DIBL [mV V^−1^]	Sidewall slope [°]
*V*_DS_ = 1V	[10]	MoS_2_	43	7.13 × 10^1^	2.46 × 10^−5^	2.90 × 10^6^	600	333	30–40
	[11]	MoS_2_	150	2 × 10^1^	1.00 × 10^−7^	2.00 × 10^8^	500	606	90
	[12]	MoS_2_	500	5.5 × 10^−1^	9.34 × 10^−7^	5.89 × 10^5^	117	126	75–80
	[13]	MoS_2_	900	2 × 10^1^	2.00 × 10^−5^	1.00 × 10^6^	600	150	80
	This Work	WS_2_	150	5.28	2.67 × 10^−8^	1.98 × 10^8^	280.6	118.8	70+10
	This Work	WS_2_	300	3.37	3.20 × 10^−9^	1.05 × 10^9^	225.3	125	70+10
	This Work	WS_2_	500	6.2 × 10^−1^	8.59 × 10^−10^	7.22 × 10^8^	192.7	111.1	70+10
	This Work	WS_2_	700	2.6 × 10^−1^	7.64 × 10^−10^	3.40 × 10^8^	157.2	72.3	70+10
*V*_DS_ = 0.1V	[10]	MoS_2_	43	5	2.94 × 10^−7^	1.70 × 10^7^	418	333	30
	[11]	MoS_2_	150	5	2.50 × 10^−6^	2.00 × 10^6^	500	606	90
	This Work	WS_2_	150	3.8 × 10^−1^	6.94 × 10^−10^	5.48 × 10^8^	170.8	118.8	70+10
	This Work	WS_2_	300	2.9 × 10^−1^	5.64 × 10^−10^	5.14 × 10^8^	149.8	125	70+10
	This Work	WS_2_	500	2.2 × 10^−2^	1.18 × 10^−9^	1.86 × 10^7^	145.9	111.1	70+10
	This Work	WS_2_	700	2.4 × 10^−2^	1.30 × 10^−9^	1.85 × 10^7^	146.8	72.3	70+10

It is evident that our devices outperform the other sidewall FETs in key performance metrics essential for low power applications.^[^ ^6^, ^41^, ^42^ ^]^ Specifically, our devices exhibit the lowest *I*_OFF_, the highest *I*_ON_/*I*_OFF_ ratio, excellent *SS*, and significantly suppressed DIBL, highlighting strong immunity to SCEs.^[^ ^43^, ^44^ ^]^ Although *I*_ON_ in our 150 nm channel length device is lower than in ref. [10]—which reported a much shorter 43 nm channel length—ref. [10] employed a relatively shallow sidewall angle of 30–40°, thereby limiting the area scalability.^[^ ^10^ ^]^ Moreover, the device in ref. [10] severely suffers from SCEs, as indicated by poor DIBL and *SS*. Compared to refs. [11] and [13], our devices demonstrate markedly improved *SS*, DIBL, and *I*_OFF_, despite slightly lower *I*_ON_.^[^ ^11^, ^13^ ^]^ In the case of ref. [12], although *I*_ON_ is comparable, our device achieves three orders of magnitude higher *I*_ON_/*I*_OFF_ ratio due to superior *I*_OFF_ suppression.^[^ ^12^ ^]^ The observed performance enhancement in our devices, including improved *SS*, reduced DIBL, and lower *I*_OFF_, primarily stems from two key process innovations. First, the dual‐step sidewall profile enables void‐free adhesion of the monolayer WS_2_ to the SiO_2_ surface. Second, the gate electrodes were directionally deposited along the sidewall using a tilted‐angle evaporation process,^[^ ^45^, ^46^ ^]^ ensuring conformal gate coverage and enhanced gate‐to‐channel coupling (Figure S7, Supporting Information). These factors together lead to stronger electrostatic control over the channel and significant suppression of short‐channel effects. These comparative results clearly validate the superiority of our vertical sidewall WS_2_ FETs for low power logic applications, demonstrating enhanced electrostatic gate control and scalability.

### 2.3 Integration of Vertical Sidewall and Planar WS_2_ FETs for Area‐Efficient Logic Circuits

**Figure 4:**
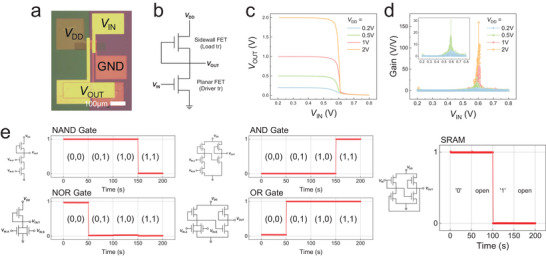
Integration of DG vertical sidewall and planar WS_2_ FETs for logic gates. a) Optical microscopy image of nMOS inverter using vertical sidewall and planar FETs. b) Circuit diagram of nMOS inverter composed of vertical sidewall FETs as the load transistor and planar FETs as the driver transistor. c) Voltage transfer characteristics of nMOS inverter measured at various *V*_DD_. d) Voltage gain extracted from (c). e) Circuit diagrams and corresponding logic operations of NAND, NOR, AND, OR logic gates, and SRAM.

Next, DG vertical sidewall FETs and planar FETs were fabricated together using CVD‐grown monolayer WS_2_ and integrated into various logic gate circuits as presented in **Figure** **4**. As illustrated in Figure 1a, employing vertical sidewall FETs in nMOS inverters leads to a dramatic reduction in the area of logic gates. If the remaining planar FETs are also replaced with vertical sidewall FETs, the integration density could be further enhanced. NMOS inverter was realized using vertical sidewall FETs as load transistor and planar FETs as the driver transistors (Figure 4b). This configuration was selected because the load transistor, with lower *V*_TH_, turns on first and holds the output voltage (*V*_OUT_) at the supply voltage (*V*_DD_), as the input voltage (*V*_IN_) increases. As the driver transistor subsequently turns on, *V*_OUT_ is pulled down to 0 V. To ensure proper inverter operation, *I*_ON_ of the load transistor should be less than that of the driver transistor.^[^ ^47^, ^48^, ^49^ ^]^ Therefore, *L*_CH_ of vertical sidewall FETs, used as the load transistor, was set to 700 nm. The voltage transfer curve (VTC) and voltage gain of the fabricated nMOS inverter are presented in Figure 4c,d. The inverter exhibits a clear voltage transition for various *V*_DD_ values, with the inversion occurring above *V*_TH_ of the driver transistor, confirming proper nMOS inverter operation.^[^ ^50^, ^51^ ^]^ Regarding voltage gain (= |∂*V*_OUT_/∂*V*_IN_|), the extracted values are 5.8, 30, 56.9, and 159.7 for *V*_DD_ = 0.2, 0.5, 1, and 2 V, respectively. Various nMOS logic gates, including NAND, NOR, AND, OR, and SRAM, were demonstrated using the nMOS inverter along with additional vertical sidewall FETs and planar FETs, as shown in Figure 4e. Optical microscopy images of each logic gate are presented in Figure S8 (Supporting Information). In all nMOS logic gates, as in the nMOS inverter, vertical sidewall FETs (*L*_CH_ = 700 nm) serve as the load transistor, while planar FETs functions as the driver transistor. NAND, NOR, AND, and OR logic gates exhibit correct functionality for all input combinations ((0,0), (0,1), (1,0), and (1,1)), thereby confirming reliable operation.^[^ ^49^, ^52^ ^]^ Similarly, in SRAM, *V*_OUT_ remains stable even after the input is held for 50 s and the circuit is opened, confirming successful data retention.^[^ ^53^, ^54^ ^]^ Moreover, all fabricated logic gates maintain stable functionality for over 200 s, validating the feasibility of integrating vertical sidewall FETs and planar FETs into area‐efficient functional circuits. Furthermore, all electrical measurements on the vertical sidewall WS_2_ FETs and integrated logic circuits were carried out under ambient conditions (room temperature, atmospheric pressure, and air atmosphere) without any encapsulation or passivation, indicating that the vertical sidewall architecture maintains stable performance without environmental degradation during the entire measurement period.

### 2.4 Performance Enhancement in Vertical and Planar Multi‐Channel WS_2_ NSFETs

**Figure 5:**
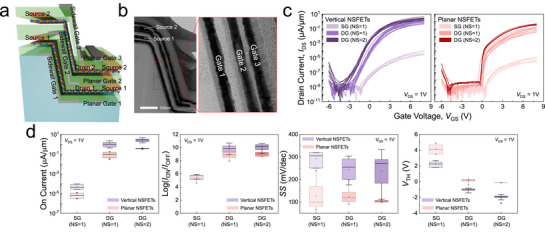
Electrical performance of vertical and planar multi‐channel WS_2_ NSFETs. a) Schematic illustration of multi‐channel WS_2_ NSFETs fabricated using vertically stacked WS_2_ channels. b) Cross‐sectional TEM image of vertical NSFETs. c) *I*_DS_–*V*_GS_ characteristics of vertical and planar multi‐channel NSFETs with different number of nanosheets and gate configurations. d) Extracted *I*_ON_, *I*_ON_/*I*_OFF_ ratio, *SS*, and *V*_TH_ for vertical and planar multi‐channel NSFETs with different number of nanosheet and gate configurations.

Since we successfully demonstrated nMOS logic gates based on vertical sidewall FETs and planar FETs, we further investigated the possible performance enhancement through the use of multiple channels.^[^ ^55^, ^56^ ^]^ As illustrated in **Figure** **5**a, vertically stacking WS_2_ channels enables the fabrication of both vertical and planar multi‐channel WS_2_ NSFETs. In multi‐channel WS_2_ NSFETs, two WS_2_ NSs are surrounded by three gates. All three gates are electrically connected, as are the two sources and the two drains, thereby allowing the device to operate as single FETs. Although multi‐channel NSFETs with planar configurations have been previously reported,^[^ ^57^, ^58^ ^]^ this study presents the first demonstration of a corresponding architecture employing vertical channels. The *L*_CH_ was 500 nm for the vertical NSFETs and 10 µm for the planar NSFETs. The cross‐sectional TEM image in Figure 5b confirms the successful fabrication of the vertical NSFETs structure. To evaluate the performance improvement achieved by vertically stacking additional channels in the vertical channel NSFETs, the *I*_DS_–*V*_GS_ characteristics at each stage of the stacking process were compared, as shown in Figure 5c. In this case, *V*_GS_ was swept from –6 to 8 V with a step of 0.1 V, and *V*_OV_ was set to 7 V. The term “single gate (SG)” refers to a configuration with only a back‐gate, without the Al seed and Al_2_O_3_‐induced n‐type doping.^[^ ^27^ ^]^ As a result, both vertical sidewall FETs and planar FETs exhibit low *I*_ON_, measured at ≈5.4 × 10^−5^ and 7.1 × 10^−6^ µA µm^−1^, respectively (Figure 5d). If the second gate is introduced to convert a SG structure into a DG structure, one WS_2_ NS becomes electrostatically controlled from both sides, leading to a substantial enhancement in *I*_ON_, reaching 1.15 µA µm^−1^ for vertical sidewall FETs and 0.10 µA µm^−1^ for planar FETs, while maintaining minimal variation in *I*_OFF_. Consequently, the *I*_ON_/*I*_OFF_ ratio significantly improves to ≈10^10^ and ≈10^9^, respectively. With the addition of one more WS_2_ channel and the third gate, the two vertical and planar channels are electrostatically modulated by three gates. This configuration provides multiple current pathways and enables enhanced drive current.^[^ ^41^, ^58^ ^]^ In Figure 5d, the median value of *I*_ON_ in the vertical and planar multi‐channel WS_2_ NSFETs increases to 2.82 and 0.38 µA µm^−1^, respectively, demonstrating maximized current drivability through vertical stacking of additional channel. The *I*_ON_/*I*_OFF_ ratio increases accordingly to ≈10^10^ and ≈10^9^, respectively. Meanwhile, both the vertical and planar multi‐channel NSFETs exhibit a reduction in *SS* with an increasing number of gates, which suggests enhanced gate control over the channels due to the adoption of a gate‐all‐around–like structure.^[^ ^59^, ^60^ ^]^ The median *SS* values of the vertical and planar multi‐channel NSFETs are 236.7 and 103.6 mV dec^−1^, respectively. Based on the device geometry and multi‐gate configuration, the electrostatic potential distribution is expected to show significantly improved gate‐to‐channel coupling, effectively enhancing channel modulation and suppressing SCEs. The strong gate control observed experimentally can be attributed to the enhanced electrostatic coupling, where multiple gates surround and simultaneously modulate the WS_2_ nanosheet channels. This multi‐gate architecture leads to superior electrostatic control, as reflected in the overall enhanced device performance, leading to superior electrostatic control and overall enhanced device performance. As a result, the introduction of additional gates facilitates more efficient current conduction at lower *V*_GS_, accompanied by a noticeable negative shift in the threshold voltage. Accordingly, *V*_TH_ exhibits a negative shift. Analysis of the trends in *I*_ON_, *I*_ON_/*I*_OFF_ ratio, *SS*, and *V*_TH_ indicates that multi‐channel NSFETs offer significant potential for further performance enhancement.

## 3 Conclusion

In this study, we demonstrated DG vertical sidewall FETs based on CVD‐grown monolayer WS_2_ and their integration into area‐efficient logic gates. By utilizing a dual‐step sidewall profile achieved through optimizing the etching condition, we obtained reliable adhesion of 2D film and enhanced gate controllability, leading to improved device yield and performance variability. The use of HfO_2_ dielectric enabled the scaling of EOT to 1.59 nm, which effectively suppressed SCEs and enhanced the *I*_ON_/*I*_OFF_ ratio and *SS* even at reduced *L*_CH_ to 150nm. Area‐efficient logic gates based on vertical sidewall FETs and planar FETs exhibited correct functionality and long‐term stability. Furthermore, we introduced a vertical multi‐channel WS_2_ NSFETs architecture, in which vertically stacked two channels and three gates configurations significantly improved the performance, demonstrating the scalability and potential of vertical channel 2D FETs for area‐efficient logic applications.

## 4 Experimental Section

### Growth and Transfer of Monolayer WS_2_

Monolayer WS_2_ was purchased from 6 Carbon Technology, where it was epitaxially grown on c‐cut sapphire via CVD. To transfer the WS_2_ onto target substrates, a conventional wet transfer technique using polymethyl methacrylate (PMMA) was employed. PMMA was spin‐coated onto the WS_2_/sapphire stack at 3000 rpm for 30 s, and the sample was then floated on a 2.15 mol L^−1^ KOH solution to release the WS_2_/PMMA film. After detachment, the film was rinsed in deionized water for 24 h before being transferred to the target substrate. The transferred samples were air‐dried at room temperature, and PMMA was removed using acetone. A post‐transfer annealing step was carried out at 150 °C for 2 h to improve adhesion.

### Fabrication of WS_2_ Vertical Sidewall and Planar FETs and Logic Circuits

To form a sidewall substrate, a 1 µm wet‐oxidized SiO_2_ substrate is dry‐etched using ICP‐RIE equipment. Detailed etching conditions are summarized in Table S1 (Supporting Information). Ti/Au (10/20 nm) was deposited on the SiO_2_‐etched surface using electron beam evaporation (EBE) to serve as the back‐gate electrode for both vertical sidewall and planar FETs. During deposition, the substrate was tilted at a 45° angle to enhance gate coupling in the sidewall configuration (Figure S7, Supporting Information). For the back gate dielectric, 20 nm of Al_2_O_3_ or 10 nm of HfO_2_ was deposited using custom‐built atomic layer deposition (ALD) equipment. Trimethylaluminum (TMA) and H_2_O were used as the precursor and reactant for Al_2_O_3_, while tetrakis(ethylmethylamino)hafnium (TEMAHf) and ozone (O_3_) were used for HfO_2_ deposition. Monolayer WS_2_ was then transferred onto the target substrate, and the channel region was patterned by ICP‐RIE with a CF_4_/Ar plasma. Subsequently, Au (30 nm) was deposited as the source/drain electrode using EBE, with the substrate tilted 45° in the opposite direction to electrically isolate the source and drain electrodes. To optimize doping of the WS_2_ channel, a 1.5 nm Al seed layer followed by a 16 nm Al_2_O_3_ encapsulation layer was applied. For DG formation, the back‐gate contact pad was opened using buffered oxide etchant (BOE), and Ti/Au (10/20 nm) was deposited as the top‐gate electrode. In the construction of logic gates, an additional process of opening the metal contact pad and wiring it with Ti/Au (10/45nm) was added.

### Fabrication of WS_2_ Vertical Sidewall and Planar NSFETs

WS_2_ vertical sidewall NSFETs and planar NSFETs were fabricated following a similar process flow as that used for vertical sidewall and planar FETs fabrication. Monolayer WS_2_ was transferred onto pre‐etched SiO_2_/first‐gate/20 nm Al_2_O_3_ dielectric stacks, followed by ICP‐RIE patterning (CF_4_/Ar plasma) to define the first nanosheet channel. Source/drain electrodes were formed by depositing Au (30 nm) using electron beam evaporation, with the substrate tilted to ensure isolation between contacts. To optimize channel doping, the devices were fabricated using the same process that included Al seed layer deposition and Al_2_O_3_ encapsulation. For DG structures, second‐gate electrodes were added by opening the back‐gate pad via BOE and depositing Ti/Au. For multi‐channel device fabrication, an additional 20 nm Al_2_O_3_ dielectric layer was deposited on top. Then, a second WS_2_ layer was transferred and patterned to define the second nanosheet channel. Before depositing the second source/drain electrodes, the first source/drain contact pads were opened to electrically connect the first and second channels, enabling them to operate as single FETs. Subsequently, a top dielectric stack (comprising an Al seed layer and Al_2_O_3_ encapsulation) was applied, and the third gate electrode was deposited to complete the device structure.

### Material and Device Characterization

Optical microscopy (OM) images were acquired using a Nikon LV100NM microscope to evaluate the optical contrast and verify device dimensions with scale bars. Cross‐sectional TEM was performed using a JEM‐F200 (JEOL) equipped with an EDS detector. TEM specimens were prepared by focused ion beam (FIB) milling using a ZEISS Crossbeam 350 system. Prior to carbon coating, a chromium layer (20nm) was deposited on the sidewall to encapsulate the DG FETs and minimize damage from Ga⁺ ion bombardment during FIB processing. Electrical characteristics were measured under ambient conditions at room temperature in the dark, using a probe station (M5VC, MS Tech) connected to a semiconductor parameter analyzer (4200A‐SCS, Keithley) with a low‐current preamplifier module. All measurements were controlled via Clarius software.

## Conflict of Interest

The authors declare no conflict of interest.

## Supporting information



Supporting Information

## Data Availability

The data that support the findings of this study are available from the corresponding author upon reasonable request.
