# Supplementary material for: Large‐Scale Implementation of Vertical Sidewall and Vertical Multi‐Channel WS2 Nanosheet Field‐Effect Transistors for Area‐Efficient Integrated Circuit
Source: Small. 2025 Sep 2;21(42):e08533. doi: 10.1002/smll.202508533 (PMC12547997; doi:10.1002/smll.202508533)
Supplement: Supplementary file 1 — Supporting Information [file SMLL-21-e08533-s001.docx]

Supporting Information

**Large-Scale Implementation of Vertical Sidewall and Vertical Multi-Channel WS_2_ Nanosheet Field-Effect Transistors for Area-Efficient Integrated Circuit**

*Jiwon Ma, Eunyeong Yang, Changwook Lee, Jisoo Seok, and Jiwon Chang^*^*

((Please insert your Supporting Information text/figures here. Please note: Supporting Display items, should be referred to as Figure S1, Equation S2, etc., in the main text…)

**Figure S1.** Comparison of SiO₂ sidewall profiles and their impact on the performance of vertical sidewall MoS₂ FETs. (a), (c), (e) Scanning electron microscope images of sidewalls etched with three different conditions. (b), (d), (f) Corresponding *I*_DS_–*V*_GS_ characteristics of MoS₂ FETs fabricated on each SiO_2_ sidewall. Only the third condition (dual-step profile) yields relatively uniform performance and effective gate control.

|  | Condition 1 | Condition 2 | Condition 3 |
| --- | --- | --- | --- |
| Flow Rate of CF_4_ (sccm) | 6 | 6 | 40 |
| Flow Rate of Ar (sccm) | 30 | 30 | 10 |
| Pressure (mTorr) | 10 | 10 | 10 |
| Source Power (W) | 200 | 100 | 50 |
| Bias Power (W) | 200 | 50 | 100 |
| Masking Material | Nickel Hard Mask | Nickel Hard Mask | Negative Photoresist |
| Etch Rate (nm/s) | 2.5 | 1.2 | 1.07 |

**Table S1.** Dry etching conditions used to optimize the SiO₂ sidewall profile. Three dry etching conditions were evaluated to optimize the SiO₂ sidewall angle and MoS₂ adhesion. Condition 3 using a negative photoresist mask resulted in dual-step profile, which enabled conformal 2D film adhesion without air gap formation, as discussed in **Figure S3**.

**Figure S2.** (a) TEM image of MoS₂ film on SiO_2_. (b) the curvature at the corner region and the actual curvature of the film. (c) The curvature-induced strain at the corner promotes air gap formation, ultimately degrading device yield and variability.

**Figure S3.** (a) Geometrical strain analysis at the top corner based on TEM images, showing minimal elastic deformation due to the large curvature radius; (b) similarly, negligible strain is observed at the bottom corner, indicating minimal elastic stress within the monolayer film. These observations confirm that the monolayer film maintains mechanical, structural, and material stability, with preserved adhesion integrity at both the top and bottom corners.

**Figure S4.** Elemental EDS mapping of DG vertical sidewall WS₂ FET. Although W and S signals were too weak to be detected in the EDS analysis, the spatial distributions of O, Al, Ti, and Au clearly confirm the proper formation of DG vertical sidewall FETs on the sidewall.

**Figure S5.** Performance of planar MoS₂ FETs for different sidewall etching conditions. *I*_ON_/*I*_OFF_ ratio and SS of planar FETs remain unaffected by the etching condition.

**Figure S6.** *I*_DS_–*V*_GS_ characteristics of DG vertical sidewall and planar WS₂ FETs at low *V*_DS_. *I*_DS_–*V*_GS_ characteristics of vertical sidewall and planar FETs with *L*_CH_ = 150–700 nm for vertical sidewall FETs and with *L*_CH_ =10 μm for planar FETs (a**)** at *V*_DS_ = 0.1 V and **(**b**)** at *V*_DS_ = 0.2 V, demonstrating lower *I*_OFF_ and improved *SS* at lower *V*_DS_ compared to *V*_DS_ = 1 V.

**Figure S7.** Schematic process for electrode formation in vertical sidewall WS₂ FETs. Sidewall gate electrodes are deposited with the substrate tilted toward the sidewall to maximize gate coupling to the vertical channel. Source and drain electrodes are deposited from the opposite direction with a reversed tilt to ensure disconnection at the sidewall.

**Figure S8.** Optical microscopy images of fabricated nMOS logic gates. NAND, NOR, AND, OR, and SRAM fabricated by integration of vertical sidewall and planar WS₂ FETs.
